# Effects of Digitization of Self-Monitoring of Blood Glucose Records Using a Mobile App and the Cloud System on Outpatient Management of Diabetes: Single-Armed Prospective Study

**DOI:** 10.2196/48019

**Published:** 2024-01-19

**Authors:** Tomoko Handa, Takeshi Onoue, Tomoko Kobayashi, Ryutaro Maeda, Keigo Mizutani, Ayana Yamagami, Tamaki Kinoshita, Yoshinori Yasuda, Shintaro Iwama, Takashi Miyata, Mariko Sugiyama, Hiroshi Takagi, Daisuke Hagiwara, Hidetaka Suga, Ryoichi Banno, Yoshinori Azuma, Takatoshi Kasai, Shuko Yoshioka, Yachiyo Kuwatsuka, Hiroshi Arima

**Affiliations:** 1 Department of Endocrinology and Diabetes Nagoya University Graduate School of Medicine Nagoya Japan; 2 Research Center of Health, Physical Fitness and Sports Nagoya University Nagoya Japan; 3 Department of Endocrinology and Diabetes Japanese Red Cross Aichi Medical Center Nagoya Daini Hospital Nagoya Japan; 4 Department of Endocrinology and Metabolism Tosei General Hospital Seto Japan; 5 Department of Advanced Medicine Nagoya University Hospital Nagoya Japan

**Keywords:** app, diabetes care, diabetes, digital intervention, digital therapeutics, glycemic control, mobile app, mHealth

## Abstract

**Background:**

In recent years, technologies promoting the digitization of self-monitoring of blood glucose (SMBG) records including app-cloud cooperation systems have emerged. Studies combining these technological interventions with support from remote health care professionals have reported improvements in glycemic control.

**Objective:**

To assess the use of an app-cloud cooperation system linked with SMBG devices in clinical settings, we evaluated its effects on outpatient management of diabetes without remote health care professional support.

**Methods:**

In this multicenter, open-label, and single-armed prospective study, 48 patients with diabetes (including type 1 and type 2) at 3 hospitals in Japan treated with insulin or glucagon-like peptide 1 receptor agonists and performing SMBG used the app-cloud cooperation system for 24 weeks. The SMBG data were automatically uploaded to the cloud via the app. The patients could check their data, and their attending physicians reviewed the data through the cloud prior to the patients’ regular visits. The primary outcome was changes in glycated hemoglobin (HbA_1c_) levels.

**Results:**

Although HbA_1c_ levels did not significantly change in all patients, the frequency of daily SMBG following applying the system was significantly increased before induction at 12 (0.60 per day, 95% CI 0.19-1.00; *P*=.002) and 24 weeks (0.43 per day, 95% CI 0.02-0.84; *P*=.04). In the subset of 21 patients whose antidiabetic medication had not been adjusted during the intervention period, a decrease in HbA_1c_ level was observed at 12 weeks (*P*=.02); however, this significant change disappeared at 24 weeks (*P*=.49). The Diabetes Treatment Satisfaction Questionnaire total score and “Q4: convenience” and “Q5: flexibility” scores significantly improved after using the system (all *P*<.05), and 72% (33/46) patients and 76% (35/46) physicians reported that the app-cloud cooperation system helped them adjust insulin doses.

**Conclusions:**

The digitization of SMBG records and sharing of the data by patients and attending physicians during face-to-face visits improved self-management in patients with diabetes.

**Trial Registration:**

Japan Registry of Clinical Trials (jRCT) jRCTs042190057; https://jrct.niph.go.jp/en-latest-detail/jRCTs042190057

## Introduction

Patients with diabetes treated with insulin or the glucagon-like peptide 1 receptor agonist (GLP-1RA) are recommended to perform self-monitoring of blood glucose (SMBG), which is covered by health insurance in Japan, to achieve and maintain blood glucose within the normal range as much as possible [1-5]. SMBG data can be useful not only in confirming hypoglycemia or hyperglycemia in real time but also in the long-term management of diabetes (adjusting insulin, diet, and exercise). On the other hand, entering SMBG data into handwritten logbooks can be time-consuming, and transcription errors (or intentional misreporting) may occur [6,7]. It is also difficult for the attending physicians to accurately assess lifestyle or therapeutic problems from the patient’s SMBG record during consultation at outpatient clinics.

With the prevalent use of the internet and smartphones, increasing evidence suggests that interventions with information and communication technology effectively enhance diabetes management [8-10]. Continuous glucose monitoring devices, which have become increasingly popular in recent years, allow patients to visualize the information on glucose levels and trends in real time on a portable receiver or a smartphone app and share these data with health care professionals (HCPs) [11-13]. Although not as common as continuous glucose monitoring, SMBG devices are becoming capable of digitizing and using data. Previous studies on SMBG have reported that self-monitoring systems with glucose meters connected wirelessly to mobile apps and web-based monitoring systems have shown improved glycemic control [14-26] and have helped patients with diabetes achieve target glycemic control with less hypoglycemia [20,21]. In these studies, information and communication technology–based self-monitoring systems provided personalized medical advice, including lifestyle-related advice from HCPs by web-based messaging [14,15,17-19,21,23-26] or telephone [16,26]. However, routine clinical practice differs from these research settings in that support from remote HCPs is limited. Furthermore, several of these studies have included participants who had never performed SMBG [17,18,20-22,25], suggesting that the effects are partly attributed to the introduction of SMBG. To apply SMBG digitization in real-world clinical practices, it is necessary to investigate its effect without remote HCP support on patients who are already performing SMBG. However, no such study has yet been conducted to date.

In recent years, several app-cloud cooperation systems that use cloud-computing services and mobile apps linked to SMBG devices have been used by patients with diabetes in Japan [27-29]. The apps used in these systems support patients’ lifestyles by digitization of SMBG records and visualization of blood glucose levels. These apps are also linked to cloud-computing services, which allow the sharing of information registered in the app with HCPs via the internet. HCPs can easily see a patient’s recent progress and trends in blood glucose variability by referring to simple graphs and summaries. Thus, the app-cloud cooperation systems allow HCPs to monitor and analyze patients’ trends in blood glucose levels and lifestyle problems at any time. These features of the app-cloud cooperation system would be beneficial if attending physicians could analyze the data before every visit of patients, as consultation time is limited in most clinical settings. These commercially available app-cloud cooperation systems are already in use among certain patients and medical institutions in Japan, and similar systems are gaining worldwide popularity. However, prospective data validating their effectiveness are lacking.

Therefore, in this study, we used a commercially available app-cloud cooperation system that is widely used in Japan and is linked to SMBG devices and evaluated its effects on glycemic control, self-management, behavioral change, or treatment satisfaction with only feedback from the attending physician during face-to-face visits in patients with diabetes (including type 1 and type 2) treated with insulin or GLP-1RA and already performing SMBG.

## Methods

### Study Design

This was a 24-week, multicenter, open-label, and single-armed prospective study conducted at 3 participating hospitals in Japan (Nagoya University Hospital, Japan Red Cross Medical Center Nagoya Daini Hospital, and Tosei General Hospital). The trial is registered in the Japan Registry of Clinical Trials (jRCTs042190057).

### Ethical Considerations

The study protocol was approved by the ethics committee of Nagoya University Graduate School of Medicine (2019-0142) and performed in accordance with the ethical principles of the Declaration of Helsinki. All enrolled patients provided written consent to participate after they were informed of the study purpose and the potential risks and benefits. Our study guarantees the protection of privacy and confidentiality of participants by ensuring that the study data are anonymized. Participants were not provided any compensation for study participation.

### Smart e-SMBG System

**Figure 1:**
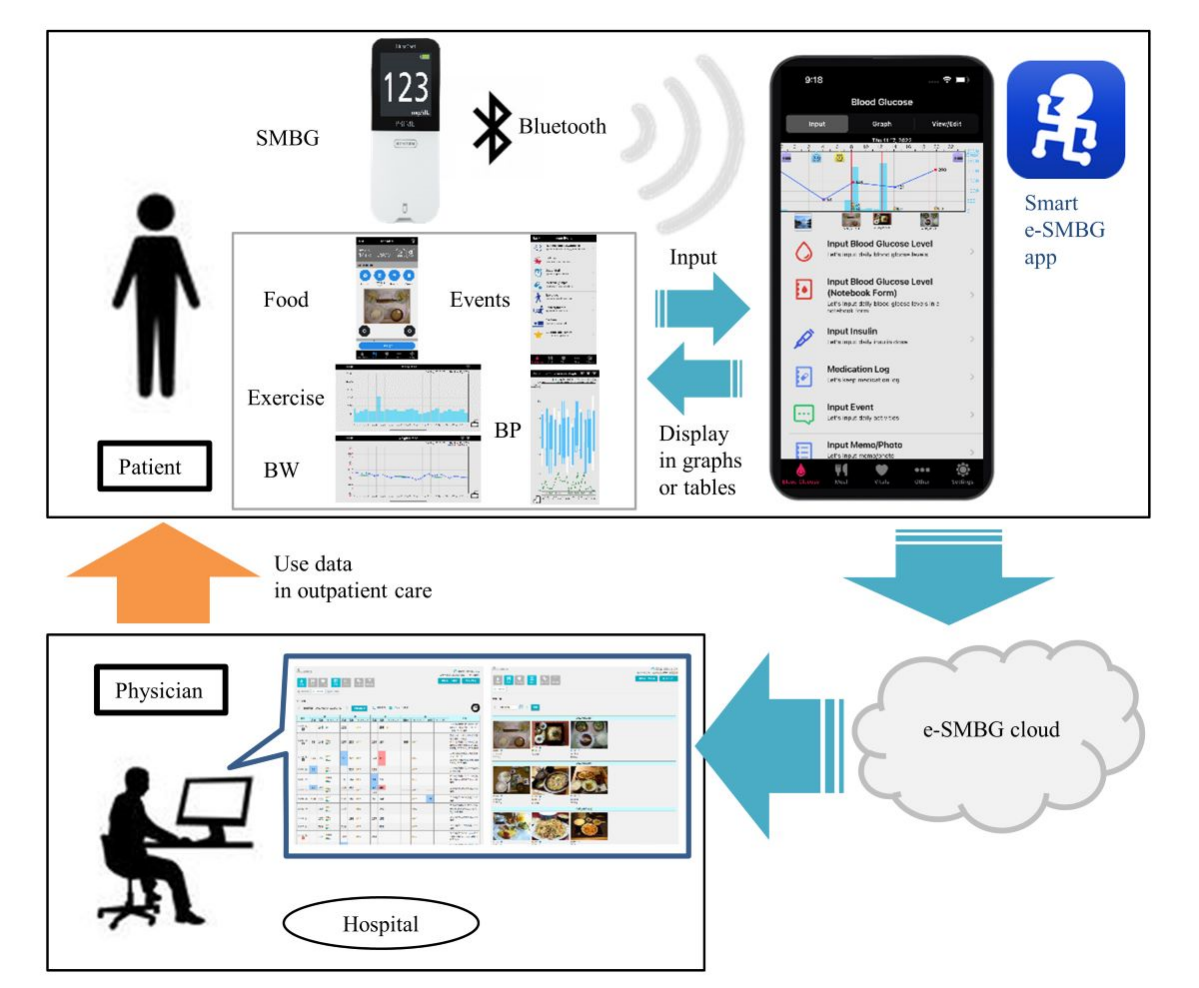
Overview of the Smart e-SMBG app and e-SMBG cloud. BP: blood pressure; BW: body weight; SMBG: self-monitoring of blood glucose.

The Smart e-SMBG system (ARKRAY, Inc) is one of the commercially available app-cloud cooperation systems for the management of diabetes using the cloud-computing service “e-SMBG Cloud” and the “Smart e-SMBG app” (for Android and iOS) linked to several SMBG devices. By linking the patient’s blood glucose meter with the Smart e-SMBG app using Bluetooth or near-field communication, the measured glucose value can be automatically transferred into the app when the patient performs an SMBG measurement. Patients can also enter health-related data such as blood pressure, weight, and step counts, as well as dietary records, treatment records, and event records, such as hypoglycemia, into this app. The entered glucose values and these data are transmitted to an e-SMBG cloud server via a wireless network. Attending physicians can review each patient’s report on the e-SMBG cloud from their office computers to use the data in outpatient care. Thus, the Smart e-SMBG system is characterized by its ability to collaborate with medical institutions and physicians. An overview of the Smart e-SMBG app and e-SMBG cloud is shown in Figure 1.

Screenshots of what the patient can see in the Smart e-SMBG app are shown in Figures S1-S4 in Multimedia Appendix 1. Specifically, patients can view the blood glucose record, including the blood glucose logbook and blood glucose variability graph (Figure S2 in Multimedia Appendix 1). Patients can also view the events, dietary and insulin records (Figure S3 in Multimedia Appendix 1), and activity and weight records (Figure S4 in Multimedia Appendix 1). Physicians can view data, such as the weekly summary, list of dietary records, and blood glucose variability graph, on the e-SMBG cloud (Figure S5 in Multimedia Appendix 1).

### Patients

Outpatients with diabetes from 3 participating hospitals were recruited. Diabetes was diagnosed based on the diagnostic criteria of the Japan Diabetes Society [30]. The inclusion and exclusion criteria for the study are detailed in Textbox 1. To accurately evaluate the effectiveness of the intervention by the app-cloud cooperation system linked to SMBG devices, we included patients who were currently performing SMBG but had no history of using a system similar to the Smart e-SMBG app and required improved glycemic control.

**Inclusion and exclusion criteria.**

**Inclusion criteria**

- Glycated hemoglobin ≥7% and <8.9% within the previous 2 months
- Patients who are currently performing self-monitoring of blood glucose
- Patients who have a smartphone or tablet for using the Smart e-SMBG app
- Patients who have not previously used the Smart e-SMBG and similar apps
- Patients who are currently using a blood glucose meter that can be linked to the Smart e-SMBG app: Glucocard G Black (GT-1830 ARKRAY, Inc), Glucocard Plus Care (GT-1840 ARKRAY, Inc), Glucotest Aqua (GT-7510 Sanwa Kagaku Kenkyusho Co, Ltd), Glucocard Prime (GT-7510 ARKRAY Inc), or Glucotest Neo Alpha (GT-1830 Sanwa Kagaku Kenkyusho Co, Ltd)
- Aged ≥20 years

**Exclusion criteria**

- Patients who cannot properly operate the devices
- Those who are judged unsuitable by their physicians for participation in the study

### Registration

Participants who qualified the above criteria and visited 1 of the 3 participating hospitals between June 24, 2019, and March 31, 2021, were eligible for recruitment.

### Intervention

After informed consent was obtained, the patients downloaded the Smart e-SMBG mobile app on iOS or Android. The patients were then instructed on how to use the app and used it in conjunction with their blood glucose meter for 24 weeks. The patients were also encouraged to enter health-related data, such as blood pressure, weight, and step counts, as well as dietary records, treatment records, and event records. The attending physician could view their patients’ data on the e-SMBG cloud and were provided with reports of blood glucose lists, a weekly summary, lists of dietary records, and blood glucose variability graphs at each regular patient regular monthly visit. The attending physician could check these reports before every visit of the patient and review them with the patient to adjust treatment and guidance.

Information on patients’ age, sex, BMI, type of diabetes, complications, and medical history were collected from electronic medical records upon enrollment. Type 1 diabetes was diagnosed based on the diagnostic criteria of the Japan Diabetes Society [31,32], whereas type 2, pancreatic, and steroid diabetes were diagnosed based on clinical data. Laboratory data, SMBG data for the past 2 weeks, and changes in diabetes medication were collected at enrollment, 12 weeks, and 24 weeks. The Diabetes Treatment Satisfaction Questionnaire (DTSQ) was used to assess patient satisfaction with the diabetes treatment [33], and the Japanese version of the DTSQ [34] was answered at enrollment, 12 weeks, and 24 weeks. The following were the items of the DTSQ: Q1=“satisfaction with current treatment,” Q2=“frequency of hyperglycemia,” Q3=“frequency of hypoglycemia,” Q4=“convenience,” Q5=“flexibility,” Q6=“understanding of diabetes,” Q7=“recommend treatment to others,” and Q8=“willingness to continue the current treatment.” Each item was assessed using a 7-point Likert scale, with scores from 0 (very dissatisfied) to 6 (very satisfied). Furthermore, a questionnaire for patients and physicians was administered at the end of the intervention.

### Outcomes

The primary outcome was the change in glycated hemoglobin (HbA_1c_) level. Secondary outcomes included changes in insulin dose, frequency of daily SMBG, DTSQ score, parameters for glycemic variability, and hypoglycemia. The parameters for glycemic variability included the SD of glucose and mean amplitude of glycemic excursions (MAGE) [35-37]. The parameters for hypoglycemia included low blood glucose index (LBGI) [38]. Treatment intensification was defined as an addition or dose increase of hypoglycemic agents, including insulin or GLP-1RA. Treatment reduction was defined as a discontinuation or dose reduction of these agents.

### Sample Size

Based on the results of a previous clinical trial [39,40], the geometric SD of the change in HbA_1c_ at the last observation period was assumed to be 0.7%. We estimated that ≥46 patients were required to confer a power of 90% to detect a 0.5% significant difference in the change from baseline at the end of the intervention. We thus planned to recruit 50 patients with consideration for potential discontinuation or dropout of the enrolled patients during the study period.

### Statistical Analysis

Continuous variables are expressed as the mean (SD), and nominal variables are expressed as frequency (%) unless stated otherwise. A linear mixed model, including the treatment period as a fixed effect, was used to compare changes in the HbA_1c_ level, insulin dose, frequency of daily SMBG, DTSQ score, mean glucose, SD of glucose, MAGE, and LBGI from baseline at 12 and 24 weeks. Effect sizes for continuous variables were calculated using the paired 2-tailed *t* test and quantified using Cohen *d*. For ordinal variables, the Wilcoxon signed-rank test was used, with the effect size represented by r=Z/√n. Analyses were conducted using 2-sided tests at a significance level of .05. SAS 9.4 software and JMP Pro 15.1.0 software (SAS Institute Inc) and Stata (version 17.0; StataCorp LLC) were used for all statistical analyses.

## Results

**Figure 2:**
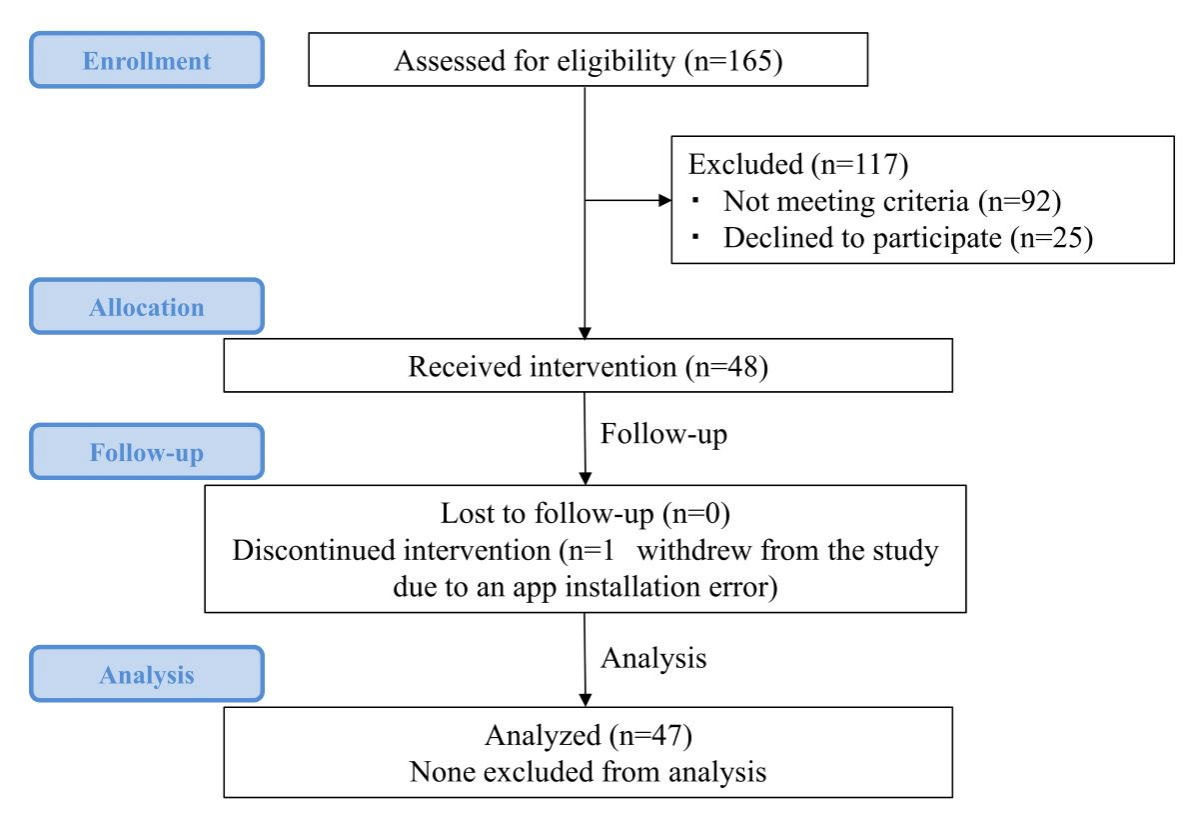
Flowchart of the study.

Figure 2 shows the CONSORT (Consolidated Standards of Reporting Trials) flow diagram of the study. In the participating hospitals, 165 candidates were assessed for eligibility for this study. Of the 165 patients, 92 did not meet the eligibility criteria and 25 patients refused to enroll in the study. The following were the reasons for the exclusion of the 92 participants: inability to properly operate the devices (n=85), anticipated difficulty in participation due to the intervals between hospital visits (n=1), poor compliance (n=2), psychiatric illness or dementia (n=3), and poor general health due to comorbidities (n=1). Therefore, 48 patients were recruited into the study. As 1 patient withdrew owing to an app installation error, 47 completed the study.

Table 1 shows the baseline characteristics of the patients. Overall, 34 patients were male and 14 were female, with a mean age of 59.8 (SD 11.9) years and a mean BMI of 25.2 (SD 4.8) kg/m^2^. The mean HbA_1c_ was 7.7% (SD 0.6%), and the mean duration of diabetes was 18.2 (SD 10.8) years. Regarding the type of diabetes, of the 48 patients, 4 (8%) had type 1 diabetes, 40 (83%) had type 2 diabetes, 3 (6%) had pancreatic diabetes, and 1 (2%) had steroid diabetes. Moreover, 31 (65%), 7 (15%), and 10 (21%) were treated with insulin only, GLP-1RA only, and both treatments, respectively.

**Table 1:** Baseline characteristics of the study patients (n=48).

Characteristic		Value
Age (years), mean (SD)		59.8 (11.9)
**Sex, n (%)**		
	Male	34 (71)
	Female	14 (29)
BMI (kg/m^2^), mean (SD)		25.2 (4.8)
HbA_1c_^a^, mean (SD)		7.7 (0.6)
Duration of diabetes (years), mean (SD)		18.2 (10.8)
**Type of diabetes, n (%)**		
	Type 1	4 (8)
	Type 2	40 (83)
	Pancreatic	3 (6)
	Steroid	1 (2)
**Type of disease, n (%)**		
	Retinopathy	22 (46)
	Nephropathy	26 (54)
	Neuropathy	19 (40)
	Cardiovascular disease	6 (13)
	Cerebrovascular disease	2 (4)
**Insulin treatment, n (%)**		
	Use of insulin	31 (65)
	Use of GLP-1RA^b^	7 (15)
	Use of both insulin and GLP-1RA	10 (21)
Insulin dose (n=41; units per day), mean (SD)		32.8 (22.4)
**Frequency of daily SMBG^c^, mean (SD)**		
	Total (n=47)	2.3 (0.9)
	MDI^d^ (n=35)	2.3 (1.0)
	Others (n=12)	2.4 (0.9)

^a^HbA1c: glycated hemoglobin.

^b^GLP1RA: glucagon-like peptide 1 receptor agonist.

^c^SMBG: self-monitoring of blood glucose.

^d^MDI: multiple daily injection.

Table 2 shows the changes in glycemic outcomes and questionnaire scores in patients. Compared to the baseline values, HbA_1c_ decreased by –0.13% at 12 weeks (*P*=.15) and –0.06% at 24 weeks (*P*=.53), but the difference was not statistically significant. The frequency of daily SMBG was significantly increased at 12 weeks (0.66 per day, 95% CI 0.25-1.07; *P*=.002) and 24 weeks (0.43 per day, 95% CI 0.02-0.84; *P*=.04). In patients on multiple daily injections, the frequency of daily SMBGs increased by 0.76 per day at 12 weeks (95% CI 0.29-1.23; *P*=.002) and 0.50 per day at 24 weeks (95% CI 0.03-0.97; *P*=.04). The MAGE (*P*=.39) and LBGI (*P*=.23) values showed a trend toward an increase after 12 weeks; however, it was not statistically significant, which may be caused by the increase in the frequency of daily SMBG. The DTSQ total score and “Q4: convenience” and “Q5: flexibility” scores were significantly improved after the use of the Smart e-SMBG app (all *P*<.05). Effect sizes for each outcome are presented in Table S1 in Multimedia Appendix 1. The average number of face-to-face visits with patients or physicians during the intervention was 4.7 (SD 1.0), and the attending physician reviewed the cloud data at every visit. No significant correlation was observed between the number of visits and HbA_1c_ change or SMBG frequency change (Table S2 in Multimedia Appendix 1).

**Table 2:** Changes in glycemic outcomes and questionnaire scores in total patients (n=47).

Parameter		Change at 12 weeks (95% CI)	*P* value	Change at 24 weeks (95% CI)	*P* value
HbA_1c_^a^ (%)		–0.13 (–0.31 to 0.05)	.15	–0.06 (–0.24 to 0.13)	.53
Insulin dose (units per day)		–1.02 (–2.53 to 0.49)	.18	–1.34 (–2.85 to 0.17)	.08
**Glycemic outcome**					
	SD of glucose (mg/dL)	3.46 (–1.80 to 8.71)	.19	0.37 (–4.93 to 5.67)	.89
	MAGE^b^ (mg/dL)	5.37 (–7.17 to 17.92)	.39	–3.04 (−15.69 to 9.62)	.63
	LBGI^c^	0.73 (–0.48 to 1.94)	.23	0.41 (–0.81 to 1.64)	.50
**Frequency of daily SMBG^d^**					
	Total (n=46)	0.66 (0.25 to 1.07)	*.002* ^*e*^	0.43 (0.02 to 0.84)	*.04*
	MDI^f^ (n=35)	0.76 (0.29 to 1.23)	*.002*	0.50 (0.03 to 0.97)	*.04*
	Others (n=11)	0.33 (–0.63 to 1.29)	.46	0.20 (–0.76 to 1.16)	.65
**DTSQ^g^ score**					
	Total score	1.74 (0.10 to 3.39)	*.04*	2.23 (0.59 to 3.87)	*.01*
	Q1: Current treatment	0.06 (–0.21 to 0.34)	.65	0.21 (–0.07 to 0.49)	.13
	Q2: Frequency of hyperglycemia	–0.13 (–0.65 to 0.39)	.62	–0.06 (–0.58 to 0.46)	.81
	Q3: Frequency of hypoglycemia	–0.13 (–0.62 to 0.36)	.60	–0.17 (–0.66 to 0.32)	.49
	Q4: Convenience	0.60 (0.10 to 1.09)	*.02*	0.74 (0.25 to 1.24)	*.004*
	Q5: Flexibility	0.49 (0.09 to 0.89)	*.02*	0.70 (0.30 to 1.10)	*.001*
	Q6: Understanding	0.32 (–0.01 to 0.65)	.06	0.32 (–0.01 to 0.65)	.06
	Q7: Recommend	0.11 (−0.38 to 0.60)	.66	0.13 (−0.36 to 0.62)	.60
	Q8: Continue	0.17 (–0.10 to 0.44)	.22	0.13 (−0.15 to 0.40)	.35

^a^HbA_1c_: glycated hemoglobin.

^b^MAGE: mean amplitude of glycemic excursion.

^c^LBGI: low blood glucose index.

^d^SMBG: self-monitoring of blood glucose.

^e^Italic formatting indicates *P* values <.05.

^f^MDI: multiple daily injection.

^g^DTSQ: Diabetes Treatment Satisfaction Questionnaire.

During the intervention period, the changes in the overall diabetes medications (insulin, GLP-1RA, and oral hypoglycemic agents) were observed as follows: at 12 weeks, treatment was continued in 28 (60%) out of 47 patients, reduced in 10 (21%), and intensified in 9 (19%); at 24 weeks, treatment was continued in 21 (45%) patients, reduced in 15 (32%), and intensified in 11 (23%).

Based on the observed medication changes in several patients, it appears that those experiencing worsening control underwent treatment intensification, whereas those showing improvement underwent treatment reduction. Therefore, to assess the effect of the intervention, post hoc subgroup analyses were performed, considering the presence or absence of treatment changes. Table 3 shows changes in glycemic outcomes and questionnaire scores in 21 patients whose antidiabetic medication has not been adjusted by the 24-week time point. HbA_1c_ decreased significantly at 12 weeks (–0.26%, 95% CI –0.47 to –0.05; *P*=.02); however, this significant change disappeared at 24 weeks. The DTSQ total score and scores for *“*Q1: convenience,” “Q2: convenience,” “Q4: convenience,” and “Q5: flexibility” were significantly improved after the use of the Smart e-SMBG system (all *P*<.05). The results of the subgroup analysis for patients whose treatment was either intensified or reduced are presented in Tables S3 and S4 in Multimedia Appendix 1. In the subgroup with intensified treatment, a significant increase in insulin dose (*P*=.003) and MAGE (*P*=.02) at 24 weeks was noted. Conversely, the subgroup with reduced treatment showed a decrease in insulin dose (*P*=.002) and MAGE (*P*=.04) at 24 weeks. In both groups, a significant increase in the frequency of daily SMBG at 12 weeks was observed (intensified: *P*=.01; reduced: *P*=.048), whereas no significant changes in HbA_1c_ levels were noted (both *P*>.05). The effect sizes for each outcome within each subgroup are presented in Tables S5-S7 in Multimedia Appendix 1.

**Table 3:** Changes in glycemic outcomes and questionnaire scores in patients whose antidiabetic medication had not been adjusted during the study (n=21).

Parameter			Change at 12 weeks (95% CI)		*P* value		Change at 24 weeks (95% CI)		*P* value
HbA_1c_^a^ (%)			–0.26 (–0.47 to –0.05)		*.02* ^b^		–0.07 (–0.28 to 0.14)		.49
**Glycemic outcome**									
	SD of glucose (mg/dL)	0.54 (–7.44 to 8.52)		.89		0.64 (–7.34 to 8.61)		.87	
	MAGE^c^ (mg/dL)	4.33 (–12.43 to 21.08)		.60		–0.31 (–17.06 to 16.45)		.97	
	LBGI^d^	0.49 (–0.47 to 1.46)		.30		–0.25 (–1.21 to 0.72)		.60	
	Frequency of daily SMBG^e^	0.31 (–0.34 to 0.97)		.33		0.25 (–0.41 to 0.91)		.44	
**DTSQ^f^ score**									
	Total score	2.33 (0.06 to 4.61)		*.04*		3.19 (0.91 to 5.47)		*.01*	
	Q1: Current treatment	0.14 (–0.26 to 0.55)		.47		0.48 (0.07 to 0.88)		*.02*	
	Q2: Frequency of hyperglycemia	0.43 (–0.20 to 1.08)		.19		0.67 (0.02 to 1.32)		*.04*	
	Q3: Frequency of hypoglycemia	–0.38 (–1.06 to 0.29)		.25		–0.52 (–1.20 to 0.15)		.12	
	Q4: Convenience	0.52 (–0.13 to 1.18)		.11		0.71 (0.06 to 1.37)		*.04*	
	Q5: Flexibility	0.33 (–0.15 to 0.82)		.17		0.67 (0.18 to 1.15)		*.01*	
	Q6: Understanding	0.24 (–0.21 to 0.69)		.28		0.19 (–0.26 to 0.64)		.39	
	Q7: Recommend	0.71 (–0.16 to 1.59)		.10		0.76 (–0.11 to 1.64)		.09	
	Q8: Continue	0.38 (–0.01 to 0.77)		.06		0.38 (–0.01 to 0.77)		.06	

^a^HbA_1c_: glycated hemoglobin.

^b^Italic formatting indicates *P* values <.05.

^c^MAGE: mean amplitude of glycemic excursion.

^d^LBGI: low blood glucose index.

^e^SMBG: self-monitoring of blood glucose.

^f^DTSQ: Diabetes Treatment Satisfaction Questionnaire.

Table 4 presents the results of the questionnaire administered to the patients and physicians after the intervention. More than 90% of the patients (44/47, 94%) and physicians (44/47, 94%) responded that the blood glucose monitoring chart (as a logbook in the SMBG format) was helpful. For the diurnal variability graphs of blood glucose, 89% (42/47) of the patients and 94% (44/47) of the physicians found them helpful. Additionally, 83% (39/47) of the patients and 77% (36/47) of the physicians reported that the Smart e-SMBG system helped motivate the patients to improve their lifestyle, and 72% (33/46) of the patients and 76% (35/46) of the physicians reported that the Smart e-SMBG system helped them with insulin dose adjustment. Furthermore, 83% (39/47) of the patients and 91% (43/47) of the physicians reported that the Smart e-SMBG system aided their diabetes treatment. In addition, 44 (96%) out of 46 patients and 45 (96%) out of 47 physicians who participated in the study indicated that they would like to continue using the Smart e-SMBG system for their diabetes care.

**Table 4:** Results of the questionnaire for patients and physicians after the intervention.

Question and response				Patients, n (%)		Physicians, n (%)
**Was the use of this e-SMBG app useful for motivating you to improve your lifestyle? (patients: n=47; physicians: n=47)**						
	Very useful			8 (17)		15 (32)
	Useful			31 (66)		21 (45)
	Not very useful			6 (13)		10 (21)
	Not useful at all			2 (4)		1 (2)
**Was the use of this e-SMBG app useful for adjusting the insulin dose? (patients: n=46; physicians: n=46)**						
	Very useful			8 (17)		18 (39)
	Useful			25 (54)		17 (37)
	Not very useful			8 (17)		11 (24)
	Not useful at all			5 (11)		0 (0)
**Was the use of this e-SMBG app useful for diabetes treatment? (patients: n=47; physicians: n=47)**						
	Very useful			7 (15)		17 (36)
	Useful			32 (68)		26 (55)
	Not very useful			6 (13)		4 (9)
	Not useful at all			2 (4)		0 (0)
**Do you want to continue to use this e-SMBG app for diabetes treatment? (patients: n=46; physicians: n=47)**						
	Yes			44 (96)		45 (96)
	No			2 (4)		2 (4)
**Did you find the following app items useful?**						
	**Blood glucose logbook (patients: n=47; physicians: n=47)**					
		Very useful	21 (45)		19 (40)	
		Useful	23 (49)		25 (53)	
		Not very useful	0 (0)		3 (6)	
		Not useful at all	3 (6)		0 (0)	
	**Blood glucose variability graph (patients: n=47; physicians: n=47)**					
		Very useful	18 (38)		20 (43)	
		Useful	24 (51)		24 (51)	
		Not very useful	4 (9)		3 (6)	
		Not useful at all	1 (2)		0 (0)	
	**Weekly summary (patients: n=46; physicians: n=45)**					
		Very useful	7 (15)		16 (36)	
		Useful	18 (39)		18 (40)	
		Not very useful	14 (30)		10 (22)	
		Not useful at all	7 (15)		1 (2)	
	**Event record (patients: n=42; physicians: n=46)**					
		Very useful	5 (12)		14 (30)	
		Useful	7 (17)		11 (24)	
		Not very useful	20 (48)		15 (33)	
		Not useful at all	10 (24)		6 (13)	
	**Dietary record (patients: n=43; physicians: n=45)**					
		Very useful	4 (9)		16 (36)	
		Useful	9 (21)		10 (22)	
		Not very useful	18 (42)		11 (24)	
		Not useful at all	12 (28)		8 (18)	
	**Blood pressure, activity, and weight records (patients: n=43; physicians: n=45)**					
		Very useful	6 (14)		16 (36)	
		Useful	11 (26)		16 (36)	
		Not very useful	15 (35)		7 (16)	
		Not useful at all	11 (26)		6 (13)	

## Discussion

### Principal Findings

Using the “Smart e-SMBG System,” an app-cloud cooperation system that supports digitization and sharing of SMBG and other health data between patients and attending physicians without special support such as remote HCP, there was a significant increase in the frequencies of SMBG and improved treatment satisfaction among patients with diabetes who performed SMBG, and there was a temporary but significant decrease in the HbA_1c_ level in the patients for whom the treatment was not changed during the study.

In this study, the digitization of SMBG records resulted in an increase in the SMBG frequency. It is possible that patients recording their blood glucose on the app and sharing their blood glucose trends with attending physicians at follow-up visits may have increased their interest in blood glucose levels. This increased attention to blood glucose levels may lead to a better understanding of specific lifestyle issues and self-improvement and improved their self-management by changing their behavior, resulting in better glycemic control. Previous studies have shown that a higher frequency of daily SMBG corresponds with better glycemic control regardless of the type of diabetes, patient’s age, or type of treatment received [16,17,20,21,41-43].

In addition to a significant increase in the total DTSQ score, there was a significant increase in the convenience and flexibility scores on the DTSQ. Using the “Smart e-SMBG system,” patients simply performed the SMBG measurement as per their usual procedure, allowing the measured data to be automatically transmitted from the blood glucose meter to the smartphone, thus reducing the need for patients to enter blood glucose data into handwritten logbooks each time. The system also offers unique features, such as weekly summaries and blood glucose level variation graphs. These features help patients manage their diabetes care more easily and flexibly, potentially contributing to both improved patient satisfaction and the low rate of dropout observed in this study. Improvement in treatment satisfaction has been shown to improve patient’s treatment compliance and promote lifestyle modifications [44]. Furthermore, attending physicians appreciated the reporting features, including a weekly summary with good visibility, with 76% (34/45) of them noting their usefulness. Such features, emphasizing convenience and simplicity, may have contributed to sustained patient-clinician interactions during the study.

Although no significant changes in HbA_1c_ levels were noted among all patients in this study, it is important to note that treatment was not fixed. This flexibility allowed the SMBG results and reports on the cloud to be used for treatment adjustments. As a result, drug therapy was intensified or decreased in some patients during the study, which may be related to the finding that there were no significant changes in HbA_1c_ in all patients. On the other hand, 72% (33/46) of the patients and 76% (35/46) of the attending physicians responded on the questionnaire that the system was useful in adjusting insulin doses, suggesting that the app-cloud cooperation system is useful for the adjustment of drug therapy. Although this is a post hoc subgroup analysis, the observed improvement in glycemic control at 12 weeks after intervention in patients in whom the treatment did not change during the study suggested that the digitization of SMBG records using the app-cloud cooperation system improved glycemic control through effects other than intensified therapy with insulin, GLP-1RA, and oral hypoglycemic agents. As indicated by the increase in the SMBG frequency, this is presumably an improvement via behavioral change. However, as no significant changes in HbA_1c_ levels were observed at 24 weeks, along with the degree of increase in the SMBG frequency attenuated at 24 weeks compared with that at 12 weeks, the long-term effects of promoting behavioral change may require further testing.

This study has demonstrated for the first time that digitization and sharing of SMBG data between patients already performing SMBG and their attending physician were useful for improving glycemic control and enhancing diabetes self-management not only for patients in limited settings with sufficient time and resources, such as research or telemedicine, but also in routine outpatient management of diabetes. The findings underscore the benefit of promoting SMBG digitization, suggesting it as a practical approach to improve self-management and treatment outcomes in diverse clinical settings for diabetes care.

### Limitations

Our study had several limitations that should be considered. First, this study had a single-armed design without a control and cannot rule out potential biases, including the Hawthorne effect, or influences from other concurrent events, including the COVID-19 pandemic. Additionally, we excluded patients who did not use smartphones or had difficulty operating the apps, which may have influenced the age and socioeconomic status of the participants. Our study group primarily consisted of participants from a specific region of Japan, which may limit the broader generalization of our findings. Furthermore, although we included patients with various diabetes types, it remains possible that there was a difference in the impact on their lifestyle modifications due to the system between patients with type 1 and type 2 diabetes. The observed improvement in HbA_1c_ levels was obtained from the post hoc subgroup analysis focusing on patients who did not change medications, and an additional evaluation of whether the behavioral changes brought about by this system led to improved glycemic control is needed with outcomes that also consider changes in medication. As the observation period of our study was limited to 24 weeks, further studies are needed to clarify whether the interaction between patients or physicians and this system continues over a long term.

### Conclusions

In conclusion, this study demonstrated that digitization of SMBG records and sharing of SMBG and other health data between patients and attending physicians and supporting the regular face-to-face visits by using the app-cloud cooperation system improved the SMBG frequency and treatment satisfaction in patients with diabetes performing SMBG. The significant outcomes achieved without the need for specialized support such as remote HCP involvement suggest the system’s potential for widespread adoption in real-world clinical practices.

## Appendix

Multimedia Appendix 1 Screenshots of the Smart e-SMBG app and additional data on effect sizes, correlation analysis, and subgroup analysis.

## Abbreviations

CONSORT: Consolidated Standards of Reporting Trial
DTSQ: Diabetes Treatment Satisfaction Questionnaire
GLP-1RA: glucagon-like peptide 1 receptor agonist
HbA1c: glycated hemoglobin
HCP: health care professional
LBGI: low blood glucose index
MAGE: mean amplitude of glycemic excursion
SMBG: self-monitoring of blood glucose
