# Supplementary material for: Effects of Digitization of Self-Monitoring of Blood Glucose Records Using a Mobile App and the Cloud System on Outpatient Management of Diabetes: Single-Armed Prospective Study
Source: JMIR Diabetes. 2024 Jan 19;9:e48019. doi: 10.2196/48019 (PMC10837757; doi:10.2196/48019)
Supplement: Multimedia Appendix 1 [file diabetes_v9i1e48019_app1.pdf]

Table of contents

Supplementary Figure S1. Screenshots of the Smart e-SMBG mobile application (for patients).

Supplementary Figure S2. Screenshots of the application's blood glucose record (for patients).

Supplementary Figure S3. Screenshots of the application's events, dietary and insulin records (for patients).

Supplementary Figure S4. Screenshots of the application's activity and weight records (for patients).

Supplementary Figure S5. Screenshots of the e-SMBG Cloud (for physicians).

Supplementary Table S1. Effect sizes of glycemic outcomes and questionnaire scores in total patients (n = 47).

Supplementary Table S2. Correlation analysis between numbers of face-to-face visits with patients/physicians during the intervention period and changes in HbA1c, frequency of daily SMBG and DTSQ total score in total patients.

Supplementary Table S3. Changes in glycemic outcomes and questionnaire scores in patients whose diabetes treatment was intensified at 24 weeks (n = 11).

Supplementary Table S4. Changes in glycemic outcomes and questionnaire scores in patients whose diabetes treatment was reduced at 24 weeks (n = 15).

Supplementary Table S5. Effect sizes of glycemic outcomes and questionnaire scores in patients whose antidiabetic medication had not been adjusted during the study (n = 21).

Supplementary Table S6. Effect sizes of glycemic outcomes and questionnaire scores in patients whose diabetes treatment was intensified at 24 weeks (n = 11).

Supplementary Table S7. Effect sizes of glycemic outcomes and questionnaire scores in patients whose diabetes treatment was reduced at 24 weeks (n = 15).

Supplementary Figure S1. Screenshots of the Smart e-SMBG mobile application (for patients).

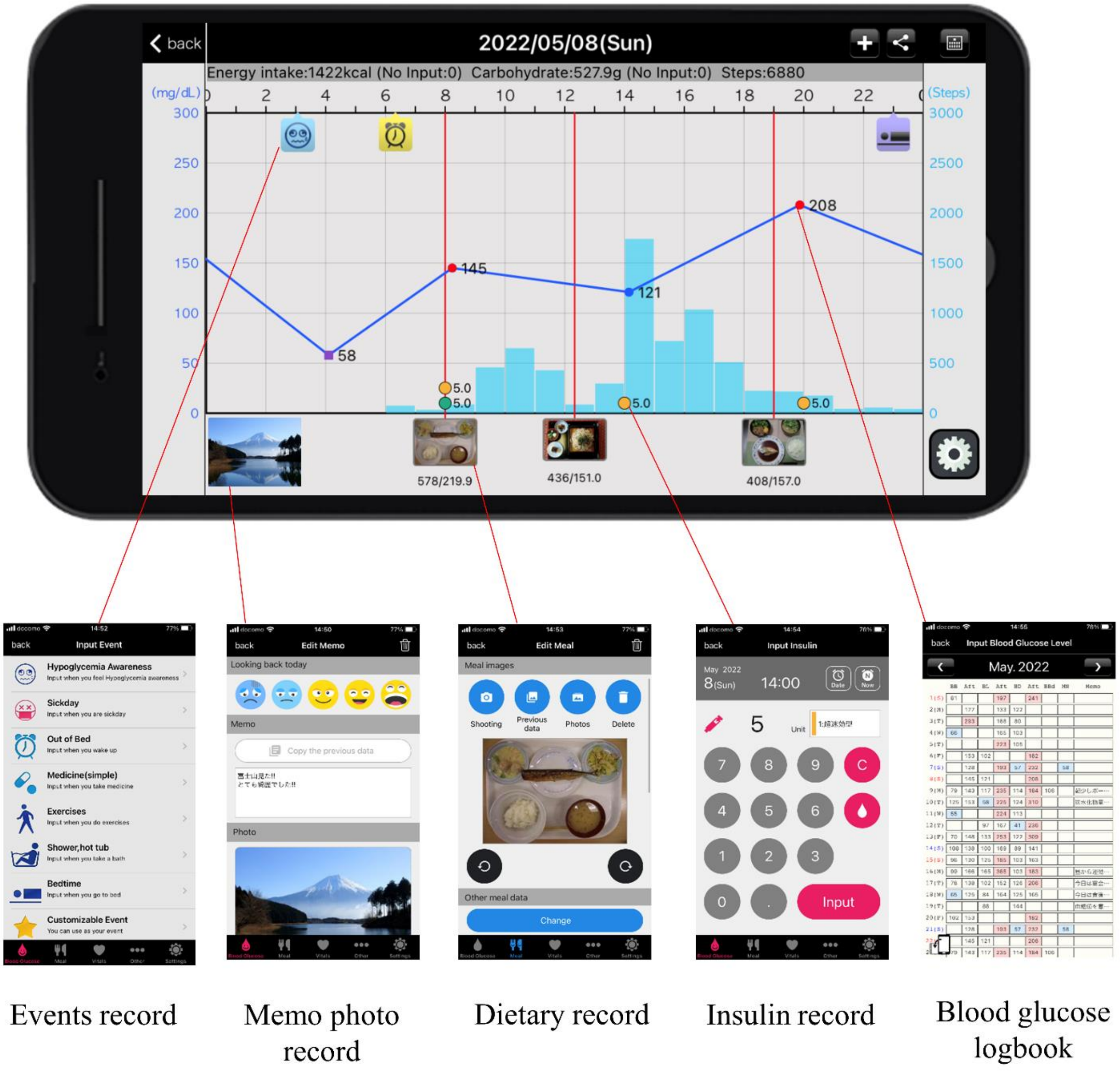

Supplementary Figure S2. Screenshots of the application's blood glucose record (for patients).

a: Blood glucose logbook, b: Blood glucose variability graph.

a.

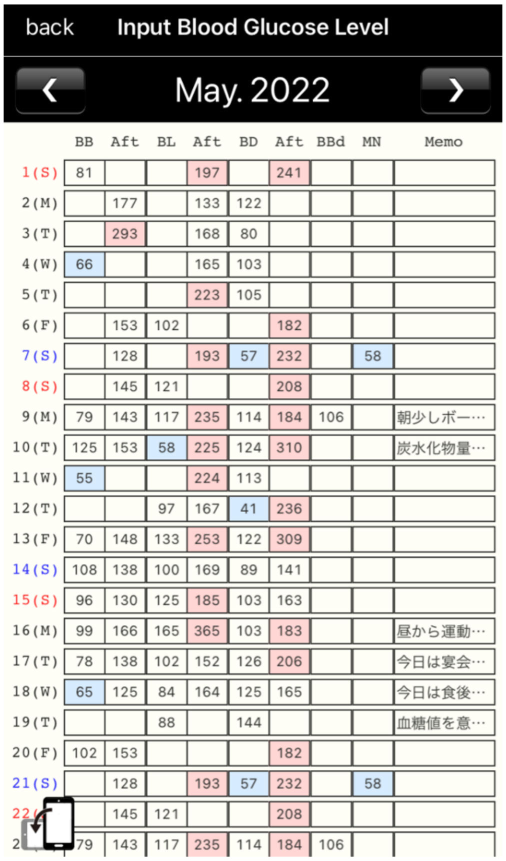

back Input Blood Glucose Level

May. 2022

	BB	Aft	BL	Aft	BD	Aft	BBd	MN	Memo
1(S)	81			197		241			
2(M)		177		133	122				
3(T)		293		168	80				
4(W)	66			165	103				
5(T)				223	105				
6(F)		153	102			182			
7(S)		128		193	57	232		58	
8(S)		145	121			208			
9(M)	79	143	117	235	114	184	106		朝少しボー…
10(T)	125	153	58	225	124	310			炭水化物量…
11(W)	55			224	113				
12(T)			97	167	41	236			
13(F)	70	148	133	253	122	309			
14(S)	108	138	100	169	89	141			
15(S)	96	130	125	185	103	163			
16(M)	99	166	165	365	103	183			昼から運動…
17(T)	78	138	102	152	126	206			今日は宴会…
18(W)	65	125	84	164	125	165			今日は食後…
19(T)			88		144				血糖値を意…
20(F)	102	153				182			
21(S)		128		193	57	232		58	
22(		145	121			208			
2	79	143	117	235	114	184	106		

b.

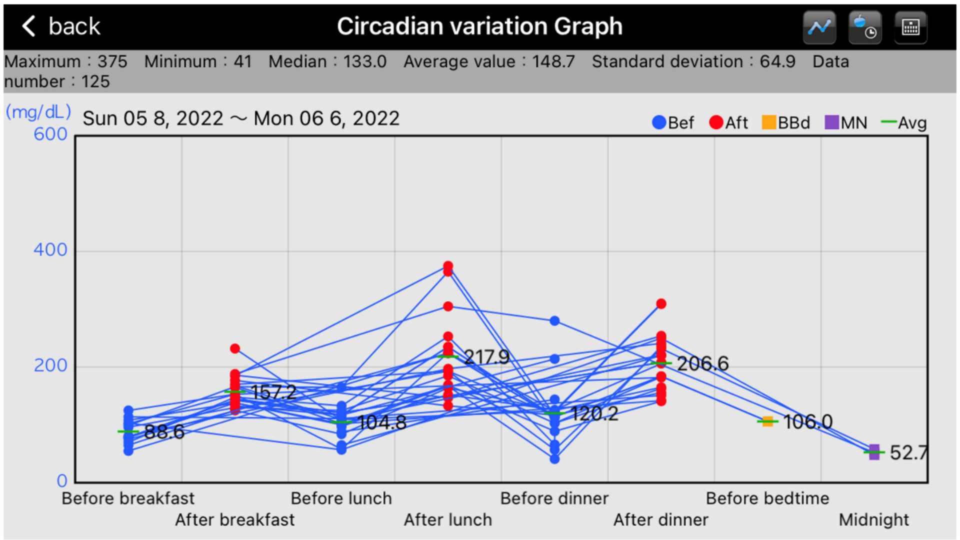

Supplementary Figure S3. Screenshots of the application's events, dietary and insulin records (for patients). a: Events record (such as awareness of hypoglycemia, sick days, and exercise); b: Dietary record (can be managed with pictures); c: Insulin record.

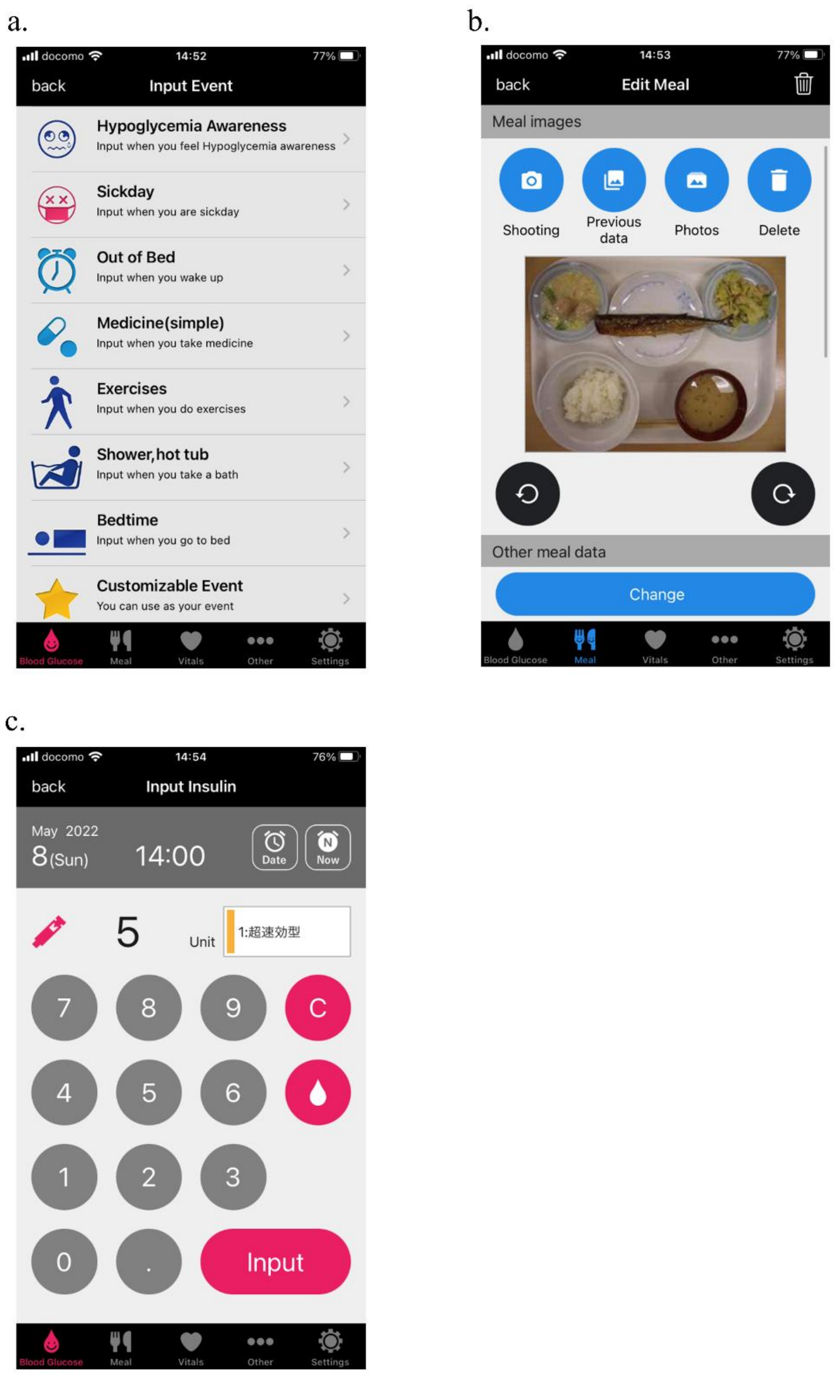

Supplementary Figure S4. **Screenshots of the application's activity and weight records (for patients).**

a: Activity record, b: Weight record

a.

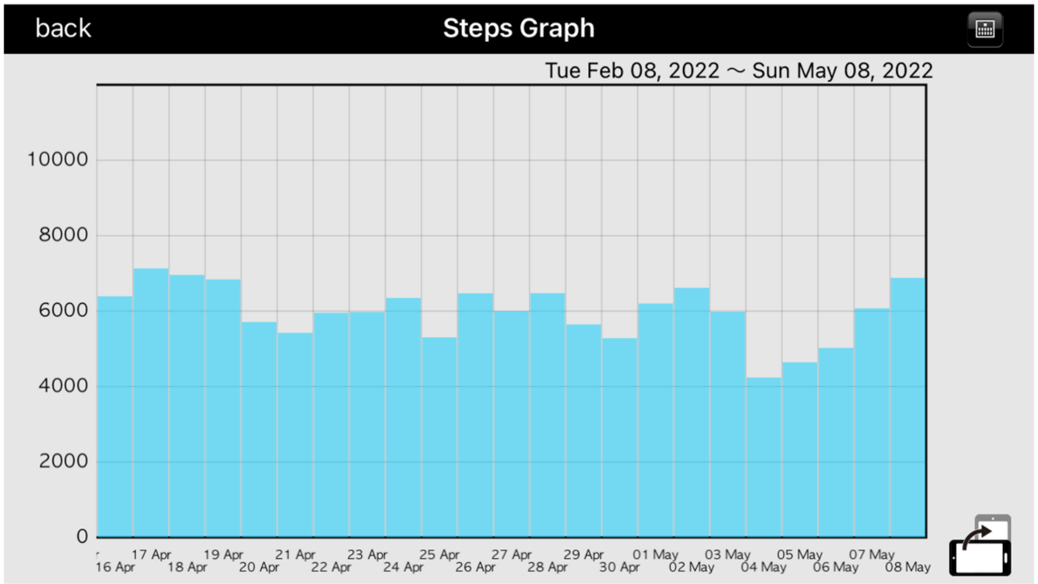

b.

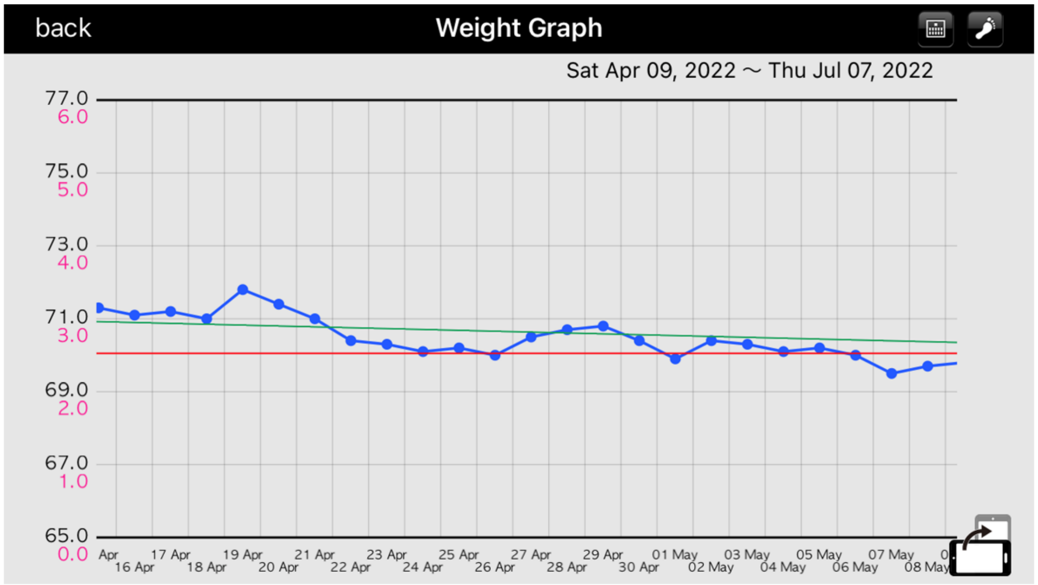

Supplementary Figure S5. Screenshots of the e-SMBG Cloud (for physicians).

a: Weekly summary, b: Blood glucose logbook, c: List of dietary records, d: Blood glucose variability graph.

a.

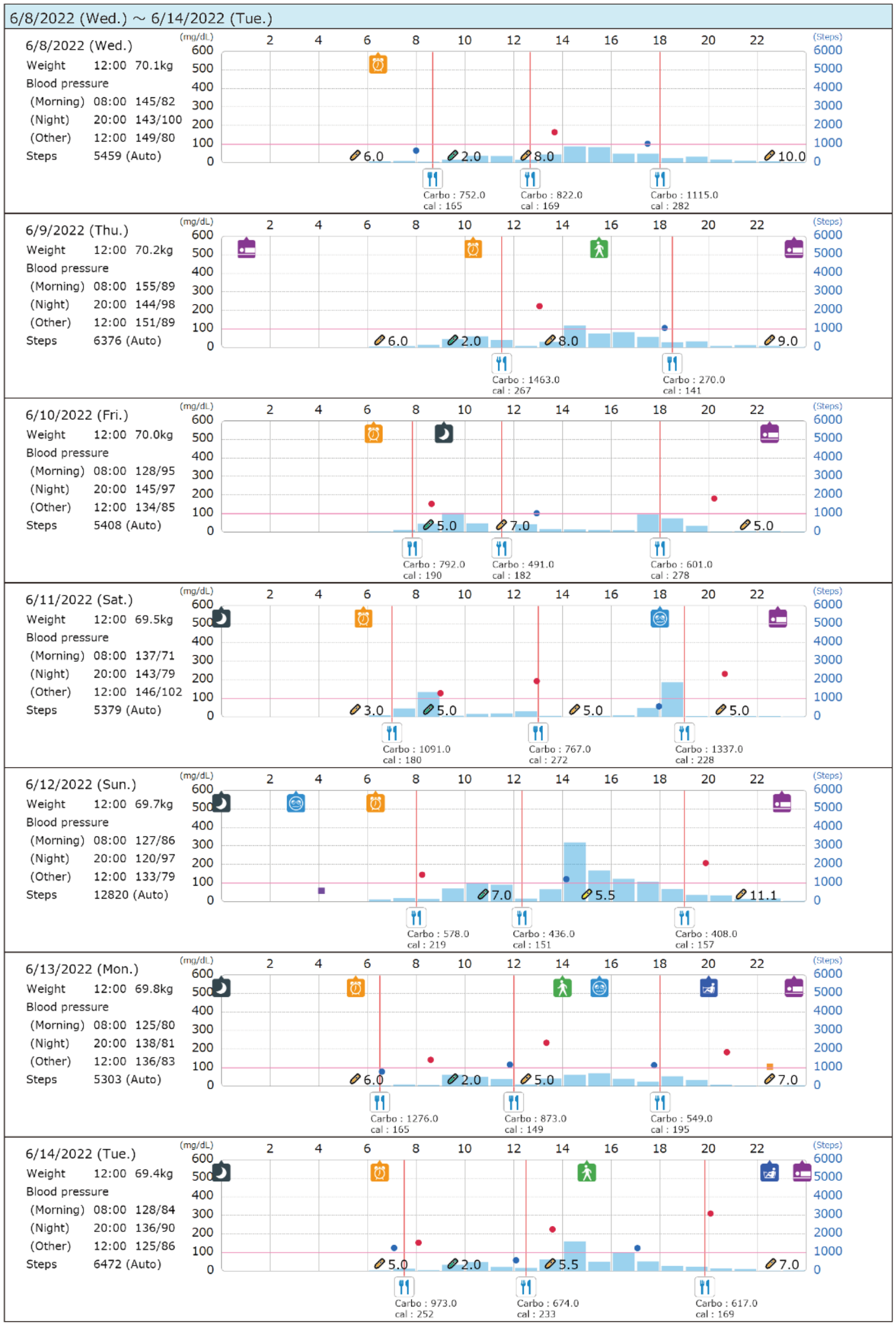

b.

Date	Morning			Daytime			Dinner			Night			
	Before meal	After meal	Insulin	Before meal	After meal	Insulin	Before meal	After meal	Insulin	Before bedtime	Insulin	Night time	Insulin
06/03 Fr		168 8:34	●6.0 ◆2.0	109 12:38		●8.0	124 20:04				●9.0		
06/04 Sa	75 7:06		◆5.0		224 13:17	●7.0	113 19:32		●5.0				
06/05 Su	81 7:40		●3.0 ◆5.0		197 13:31	●5.0		241 19:40	●5.0				
06/06 Mo		177 8:59	◆7.0		133 13:56	★5.5	122 20:39		●11.1				
06/07 Tu		293 8:43	●6.0 ◆2.0		168 13:31	●8.0	80 17:00				●9.0		
06/08 We	66 7:59		●6.0 ◆2.0		165 13:40	●8.0	103 17:29				●10.0		
06/09 Th			●6.0 ◆2.0		223 13:03	●8.0	105 18:11				●9.0		
06/10 Fr		153 8:37	◆5.0	102 12:56		●7.0		182 20:13	●5.0				
06/11 Sa		128 8:59	●3.0 ◆5.0		193 12:56	●5.0	57 17:57	232 20:39	●5.0			58 4:06	
06/12 Su		145 8:14	◆7.0	121 14:09		★5.5		208 19:52	●11.1				
06/13 Mo	79 6:35	143 8:35	●6.0 ◆2.0	117 11:50	235 13:20	●5.0	114 17:45	184 20:44		106 22:30	●7.0		
06/14 Tu	125 7:05	153 8:05	●5.0 ◆2.0	58 12:05	225 13:35	●5.5	124 17:04	310 20:04			●7.0		
06/15 We	55 7:06		●5.0 ◆5.0		224 13:17	●5.0	113 18:32				●5.0		
06/16 Th			●10.0◆5.0	97 11:31	167 14:31	●5.0	41 17:40	236 19:40			●5.0		
06/17 Fr	70 6:59	148 8:59	●6.0 ◆2.0	133 11:56	253 13:56	●8.0	122 19:39	309 20:57			●10.0		
06/18 Sa	108 7:43	138 9:43	●6.0 ◆2.0	100 11:31	169 13:31	●8.0	89 17:00	141 19:00			●9.0	52 4:06	
06/19 Su		165 8:37	●6.0 ◆2.0	102 12:56		●5.0		142 20:13			●7.0		
06/20 Mo		170 8:59	●5.0 ◆2.0		193 12:56	●5.5	117 17:57	156 20:39			●7.0		
06/21 Tu		232 8:14	●5.0 ◆5.0	108 14:09		●5.0		221 19:52			●5.0		
06/22 We		133 8:35	●10.0◆5.0	117 12:38		●5.0	214 19:53				●5.0		
06/23 Th			●6.0 ◆2.0	65 12:40		●8.0		220 20:26			●10.0		
06/24 Fr		168 8:34	●6.0 ◆2.0	109 12:38		●8.0	124 20:04				●9.0		
06/25 Sa	75 7:06		●6.0 ◆2.0		224 13:17	●5.0	113 19:32				●7.0		
06/26 Su	81 7:40		●5.0 ◆2.0		197 13:31	●5.5		241 19:40			●7.0		
06/27 Mo		177 8:59	●5.0 ◆5.0		133 13:56	●5.0	122 20:39				●5.0		
06/28 Tu		293 8:43	●10.0◆5.0		168 13:31	●5.0	80 17:00				●5.0		
06/29 We	66 7:59		●6.0 ◆2.0		165 13:40	●8.0	103 17:29				●10.0		
06/30 Th			●6.0 ◆2.0		223 13:03	●8.0	105 18:11				●9.0		
07/01 Fr		153 8:37	◆2.0 ●6.0	102 12:56		●5.0		182 20:13	●7.0				
07/02 Sa	46 10:02	128 8:59	◆2.0 ●5.0		193 12:56	●5.5	57 17:57	232 20:39	●7.0			58 4:06	
07/03 Su		145 8:14	◆5.0 ●5.0	121 14:09		●5.0		208 19:52	●5.0				

	Morning		Daytime		Dinner		Night				
	Before meal	After meal	Before meal	After meal	Before meal	After meal	Before bedtime	Night time	All pre-dinner	All postprandial	All data
Maximum	125	293	133	253	214	310	106	58	214	310	310
Minimum	46	128	58	133	41	141	106	52	41	128	41
Median	75	153	108	193	113	220	106	58	103	182	133
Average line	77	170	104	194	106	214	106	56	96	192	146
SD	21	48	20	33	34	49	0	4	30	46	62
n	12	20	15	21	22	17	1	3	53	58	111

Breakfast : 05:00～ Lunch : 11:00～ Dinner : 16:00～ Before bedtime : 22:00～ Night time : 01:00～

(The above time zone shows the time zone setting of e-SMBG Cloud. If you manually enter the time zone,the blood glucose level will be displayed in that time zone.)

● : 超速効型 ★ : 速効型 ◆ : 持効型

c.

2022/07/01 (Fri.)

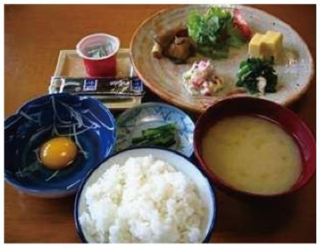
07:50
792 kcal
190.0 g

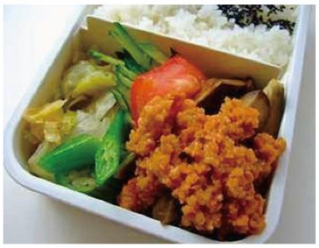
11:30
491 kcal
182.0 g

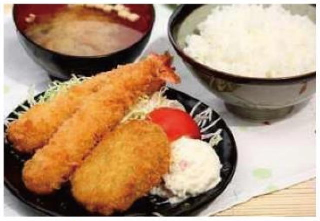
18:00
601 kcal
278.0 g

2022/07/02 (Sat.)

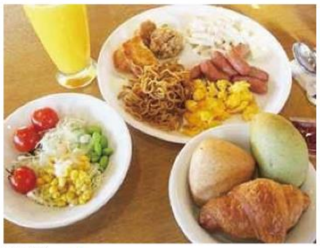
07:00
1091 kcal
180.0 g

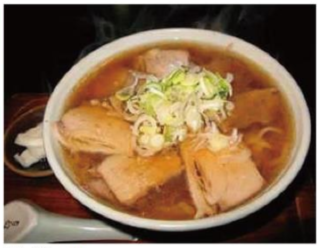
13:00
767 kcal
272.0 g

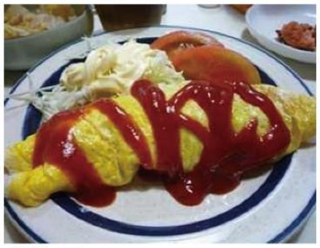
19:00
1337 kcal
228.0 g

2022/07/03 (Sun.)

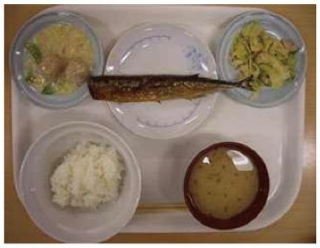
08:00
578 kcal
219.0 g

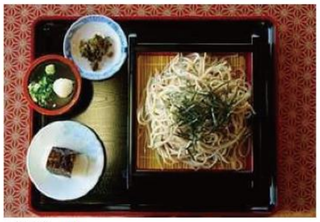
12:20
436 kcal
151.0 g

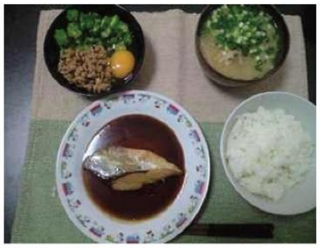
19:00
408 kcal
157.0 g

■ Number of meal data registrations(Period covered : 30 days)

	Meal	Meal + Alcohol	Snack	Fruits	Alcohol	Nutrition-supplementing food	Skip food	Total
Period registration number	79	0	0	0	0	0	0	79
Average daily	2.6	0	0	0	0	0	0	2.6

d.

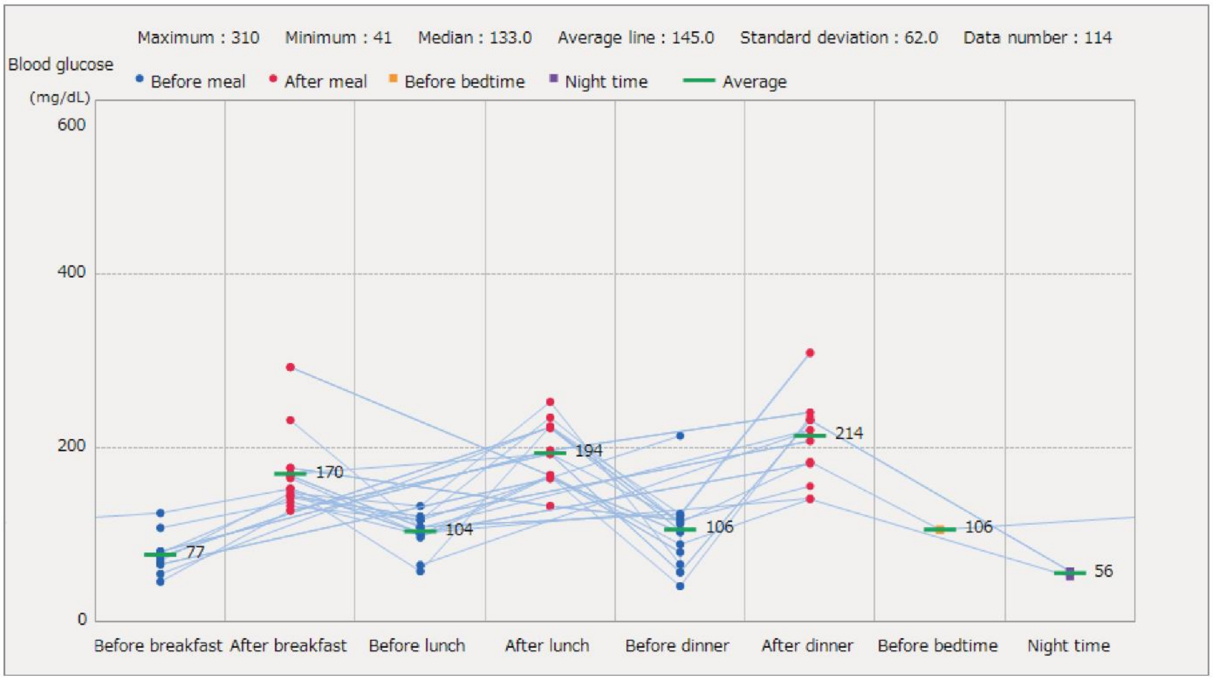
Maximum : 310
Minimum : 41
Median : 133.0
Average line : 145.0
Standard deviation : 62.0
Data number : 114
Blood glucose (mg/dL)
Before meal
After meal
Before bedtime
Night time
Average
600
400
200
0
77
170
104
194
106
214
106
56
Before breakfast
After breakfast
Before lunch
After lunch
Before dinner
After dinner
Before bedtime
Night time

Supplementary Table S1. Effect sizes of glycemic outcomes and questionnaire scores in total patients (n = 47).

	Change at 12 weeks (95% CI)	*P*-value	Cohen's d (95% CI)	Change at 24 weeks (95% CI)	*P*-value	Cohen's d (95% CI)
HbA1c (%)	−0.13 (−0.30 to 0.04)	.13	−0.19 (−0.60 to 0.21)	−0.06 (−0.25 to 0.14)	.55	−0.08 (−0.48 to 0.33)
Insulin dose (Units per day)	−1.02 (−2.45 to 0.41)	.16	−0.04 (−0.45 to 0.36)	−1.34 (−2.92 to 0.24)	.10	−0.06 (−0.46 to 0.35)
**Glycemic outcome**						
SD of glucose (mg/dL)	3.46 (−1.48 to 8.39)	.17	0.16 (−0.25 to 0.56)	0.37 (−5.27 to 6.00)	.90	0.05 (−0.36 to 0.46)
MAGE (mg/dL)	5.37 (−6.84 to 17.57)	.38	0.12 (−0.29 to 0.53)	−2.81 (−15.85 to 10.23)	.67	−0.04 (−0.45 to 0.37)
LBGI	0.72 (−0.41 to 1.86)	.21	0.21 (−0.20 to 0.61)	0.41 (−0.89 to 1.71)	.53	0.11 (−0.30 to 0.52)
**Frequency of daily SMBG**						
Total [a]	0. 60 (0.13 to 1.07)	**.01**	0.43 (0.02 to 0.84)	0.43 (0.09 to 0.78)	**.001**	0.39 (−0.02 to 0.80)
MDIs [b]	0.76 (0.22 to 1.30)	**.01**	0.54 (0.06 to 1.02)	0.50 (0.11 to 0.89)	**.01**	0.46 (−0.02 to 0.93)
Others [c]	0.11 (−0.96 to 1.18)	.82	0.09 (−0.71 to 0.89)	0.20 (−0.64 to 1.05)	.61	0.16 (−0.66 to 0.98)
DTSQ Total score	1.74 (0.25 to 3.24)	**.02**	0.32 (−0.09 to 0.72)	2.23 (0.46 to 4.01)	**.02**	0.41 (0.00 to 0.82)
**DTSQ score**	Change at 12 weeks median (IQR)	*P*-value	*r*	Change at 24 weeks median (IQR)	*P*-value	*r*
Q1 Current treatment	0.00 (0.00 to 1.00)	.59	0.08	0.00 (0.00 to 1.00)	.15	0.21
Q2 Frequency of hyperglycemia	0.00 (−1.00 to 1.00)	.80	0.04	0.00 (−1.00 to 1.00)	.97	−0.01
Q3 Frequency of hypoglycemia	0.00 (−1.00 to 1.00)	.61	−0.08	0.00 (−1.00 to 1.00)	.22	−0.18
Q4 Convenience	0.00 (0.00 to 1.00)	**.03**	0.32	0.00 (0.00 to 2.00)	**.01**	0.38
Q5 Flexibility	0.00 (0.00 to 1.00)	**.01**	0.38	1.00 (0.00 to 2.00)	**.001**	0.46
Q6 Understanding	0.00 (0.00 to 1.00)	**.049**	0.29	0.00 (−1.00 to 1.00)	.12	0.23
Q7 Recommend	0.00 (−1.00 to 1.00)	.97	−0.01	0.00 (−1.00 to 1.00)	.64	0.07
Q8 Continue	0.00 (0.00 to 0.00)	.29	0.16	0.00 (0.00 to 1.00)	.32	0.14

CI, confidence interval; SD, standard deviation; MAGE, mean amplitude of glycemic excursions; LBGI, low blood glucose index; SMBG, self-monitoring of blood glucose; MDI, multiple daily injections; DTSQ, Diabetes Treatment Satisfaction Questionnaire. *P*-values <.05 are shown in bold. [a] n = 46 [b] n = 35 [c] n = 11

Supplementary Table S2. Correlation analysis between numbers of face-to-face visits with patients/physicians during the intervention period and changes in HbA1c, frequency of daily SMBG and DTSQ total score in total patients.

	Average	SD	r	*P*-value	n
Numbers of face-to-face visits with patients/physicians (times)	4.7	1.0	0.04	.77	47
Change in HbA1c at 12 weeks (%)	−0.1	0.6			
Numbers of face-to-face visits with patients/physicians (times)	4.7	1.0	−0.17	.27	47
Change in HbA1c at 24 weeks (%)	−0.1	0.7			
Numbers of face-to-face visits with patients/physicians (times)	4.7	1.0	0.20	.19	47
Change in frequency of daily SMBG at 12 weeks (per day)	0.6	1.6			
Numbers of face-to-face visits with patients/physicians (times)	4.6	1.0	0.07	.66	46
Change in frequency of daily SMBG at 24 weeks (per day)	0.4	1.2			
Numbers of face-to-face visits with patients/physicians (times)	4.7	1.0	0.05	.71	47
Change in DTSQ total score at 12 weeks	1.7	5.1			
Numbers of face-to-face visits with patients/physicians (times)	4.7	1.0	0.06	.69	47
Change in DTSQ total score at 24 weeks	2.2	6.1			

SMBG, self-monitoring of blood glucose; DTSQ, Diabetes Treatment Satisfaction Questionnaire; SD, standard deviation. P-values <.05 are shown in bold.

Supplementary Table S3. Changes in glycemic outcomes and questionnaire scores in patients whose diabetes treatment was intensified at 24 weeks (n = 11).

	Change at 12 weeks (95% CI)	*P*-value	Change at 24 weeks (95% CI)	*P*-value
HbA1c (%)	−0.06 (−0.56 to 0.43)	.78	0.11 (−0.39 to 0.60)	.64
Insulin dose (Units per day)	1.09 (−0.06 to 2.24)	.06	2.00 (0.85 to 3.15)	**.003**
**Glycemic outcome**				
SD of glucose (mg/dL)	7.19 (−2.55 to 16.93)	.13	7.63 (−2.57 to 17.84)	.13
MAGE (mg/dL)	19.62 (−3.73 to 42.98)	.09	30.43 (5.95 to 54.91)	**.02**
LBGI	0.24 (−3.62 to 4.10)	.89	2.46 (−1.58 to 6.51)	.20
Frequency of daily SMBG [a]	1.19 (0.36 to 2.02)	**.01**	0.72 (−0.10 to 1.56)	.08
**DTSQ score**				
Total score	3.55 (−1.79 to 8.88)	.17	3.18 (−2.16 to 8.52)	.21
Q1 Current treatment	0.36 (−0.47 to 1.20)	.36	0.36 (−0.47 to 1.20)	.36
Q2 Frequency of hyperglycemia	−0.09 (−1.43 to 1.25)	.88	−0.27 (−1.61 to 1.06)	.66
Q3 Frequency of hypoglycemia	0.73 (−0.43 to 1.88)	.19	0.09 (−1.06 to 1.25)	.86
Q4 Convenience	1.36 (−0.04 to 2.76)	.06	1.27 (−0.13 to 2.67)	.07
Q5 Flexibility	1.09 (−0.23 to 2.41)	.10	1.00 (−0.32 to 2.32)	.12
Q6 Understanding	0.64 (−0.44 to 1.71)	.22	0.64 (−0.44 to 1.71)	.22
Q7 Recommend	−0.09 (−0.74 to 0.56)	.76	−0.18 (−0.83 to 0.47)	.55
Q8 Continue	0.18 (−0.65 to 1.01)	.64	0.09 (−0.74 to 0.92)	.81

CI, confidence interval; SD, standard deviation; MAGE, mean amplitude of glycemic excursions; LBGI, low blood glucose index; SMBG, self-monitoring of blood glucose; DTSQ, Diabetes Treatment Satisfaction Questionnaire. *P*-values < .05 are shown in bold.

[a] n = 10

Supplementary Table S4. Changes in glycemic outcomes and questionnaire scores in patients whose diabetes treatment was reduced at 24 weeks (n = 15).

	Change at 12 weeks (95% CI)	*P*-value	Change at 24 weeks (95% CI)	*P*-value
HbA1c (%)	0.00 (−0.40 to 0.40)	1.00	−0.16 (−0.56 to 0.24)	.41
Insulin dose (Units per day)	−2.00 (−4.07 to 0.07)	.06	−3.60 (−5.67 to −1.53)	**.002**
**Glycemic outcome**				
SD of glucose (mg/dL)	4.60 (−6.70 to 15.91)	.40	−4.88 (−16.18 to 6.42)	.37
MAGE (mg/dL)	−3.71 (−30.17 to 22.75)	.77	−28.04 (−54.50 to −1.58)	**.04**
LBGI	1.39 (−1.28 to 4.06)	.28	−0.08 (−2.75 to 2.59)	.95
Frequency of daily SMBG	0.79 (0.01 to 1.56)	**.048**	0.49 (−0.29 to 1.27)	.20
**DTSQ score**				
Total score	−0.40 (−2.51 to 1.71)	.69	0.20 (−1.91 to 2.31)	.84
Q1 Current treatment	−0.27 (−0.63 to 0.09)	.14	−0.27 (−0.63 to 0.09)	.14
Q2 Frequency of hyperglycemia	−0.93 (−1.91 to 0.05)	.06	−0.93 (−1.91 to 0.05)	.06
Q3 Frequency of hypoglycemia	−0.40 (−1.41 to 0.61)	.41	0.13 (−0.88 to 1.14)	.78
Q4 Convenience	0.13 (−0.78 to 1.04)	.76	0.40 (−0.51 to 1.31)	.36
Q5 Flexibility	0.27 (−0.40 to 0.93)	.41	0.53 (−0.13 to 1.20)	.11
Q6 Understanding	0.20 (−0.31 to 0.71)	.41	0.27 (−0.24 to 0.77)	.28
Q7 Recommend	−0.60 (−1.39 to 0.19)	.13	−0.53 (−1.33 to 0.26)	.17
Q8 Continue	−0.13 (−0.53 to 0.26)	.48	−0.20 (−0.59 to 0.19)	.29

CI, confidence interval; SD, standard deviation; MAGE, mean amplitude of glycemic excursions; LBGI, low blood glucose index; SMBG, self-monitoring of blood glucose; DTSQ, Diabetes Treatment Satisfaction Questionnaire. *P*-values < .05 are shown in bold.

Supplementary Table S5. Effect sizes of glycemic outcomes and questionnaire scores in patients whose antidiabetic medication had not been adjusted during the study (n = 21).

	Change at 12 weeks (95% CI)	*P*-value	Cohen's d (95% CI)	Change at 24 weeks (95% CI)	*P*-value	Cohen's d (95% CI)
HbA1c (%)	−0.26 (−0.44 to −0.08)	**.01**	−0.49 (−1.10 to 0.13)	−0.07 (−0.31 to 0.17)	.54	−0.12 (−0.72 to 0.49)
Insulin dose (Units per day)	−1.43 (−4.41 to 1.55)	.33	−0.06 (−0.67 to 0.54)	−1.48 (−4.45 to 1.50)	.31	−0.06 (−0.67 to 0.54)
**Glycemic outcome** [b]						
SD of glucose (mg/dL)	0.54 (−6.87 to 7.95)	.88	0.05 (−0.56 to 0.67)	0.64 (−7.87 to 9.14)	.88	0.06 (−0.55 to 0.67)
MAGE (mg/dL)	4.33 (−13.38 to 22.04)	.62	0.14 (−0.47 to 0.75)	−0.31 (−16.05 to 15.44)	.97	0.03 (−0.58 to 0.64)
LBGI	0.49 (−0.68 to 1.67)	.39	0.20 (−0.42 to 0.81)	−0.25 (−0.94 to 0.45)	.47	−0.18 (−0.79 to 0.43)
Frequency of daily SMBG	0.31 (−0.39 to 1.02)	.36	0.26 (−0.35 to 0.87)	0.25 (−0.36 to 0.86)	.41	0.23 (−0.38 to 0.83)
DTSQ Total score	2.33 (0.12 to 4.55)	**.04**	0.53 (−0.09 to 1.14)	3.19 (0.85 to 5.53)	**.01**	0.72 (0.09 to 1.34)
**DTSQ score**	Change at 12 weeks median (IQR)	*P*-value	*r*	Change at 24 weeks median (IQR)	*P*-value	*r*
Q1 Current treatment	0.00 (−1.00 to 1.00)	.51	0.14	0.00 (0.00 to 1.00)	**.01**	0.59
Q2 Frequency of hyperglycemia	1.00 (0.00 to 1.00)	.12	0.34	1.00 (0.00 to 2.00)	.06	0.42
Q3 Frequency of hypoglycemia	0.00 (−1.00 to 0.00)	.34	−0.21	0.00 (−1.00 to 0.00)	.12	−0.34
Q4 Convenience	0.00 (0.00 to 1.00)	.10	0.36	0.00 (0.00 to 2.00)	.06	0.41
Q5 Flexibility	0.00 (0.00 to 1.00)	.15	0.32	1.00 (0.00 to 1.00)	**.02**	0.51
Q6 Understanding	0.00 (0.00 to 1.00)	.22	0.27	0.00 (−1.00 to 1.00)	.50	0.15
Q7 Recommend	0.00 (−1.00 to 2.00)	.14	0.32	0.00 (0.00 to 2.00)	.11	0.34
Q8 Continue	0.00 (0.00 to 1.00)	.05	0.43	0.00 (0.00 to 1.00)	.08	0.38

CI, confidence interval; SD, standard deviation; MAGE, mean amplitude of glycemic excursions; LBGI, low blood glucose index; SMBG, self-monitoring of blood glucose; DTSQ, Diabetes Treatment Satisfaction Questionnaire. *P*-values <.05 are shown in bold.

Supplementary Table S6. Effect sizes of glycemic outcomes and questionnaire scores in patients whose diabetes treatment was intensified at 24 weeks (n = 11).

	Change at 12 weeks (95% CI)	*P*-value	Cohen's d (95% CI)	Change at 24 weeks (95% CI)	*P*-value	Cohen's d (95% CI)
HbA1c (%)	−0.06 (−0.47 to 0.34)	.73	−0.08 (−0.92 to 0.75)	−0.11 (−0.46 to 0.68)	.68	0.14 (−0.70 to 0.98)
Insulin dose (Units per day)	1.09 (0.17 to 2.01)	**.03**	0.04 (−0.08 to 0.87)	2.00 (0.66 to 3.34)	**.01**	0.06 (−0.77 to 0.90)
**Glycemic outcome** [a]						
SD of glucose (mg/dL)	7.19 (−1.43 to 15.81)	.09	0.44 (−0.41 to 1.29)	7.70 (−3.57 to 18.96)	.16	0.57 (−0.31 to 1.44)
MAGE (mg/dL)	19.62 (−1.93 to 41.17)	.07	0.61 (−0.25 to 1.46)	30.03 (3.93 to 56.12)	**.03**	1.03 (0.10 to 1.93)
LBGI	0.24 (−0.26 to 0.74)	.32	0.29 (−0.55 to 1.13)	2.46 (−3.39 to 8.32)	.37	0.44 (−0.43 to 1.31)
Frequency of daily SMBG [a]	0.88 (−0.24 to 2.00)	.11	0.61 (−0.26 to 1.46)	0.73 (0.08 to 1.38)	**.03**	0.64 (−0.25 to 1.51)
DTSQ Total score	3.55 (−1.02 to 8.12)	.12	0.61 (−0.25 to 1.46)	3.18 (−2.83 to 9.19)	.27	0.51 (−0.35 to 1.35)
**DTSQ score**	Change at 12 weeks median (IQR)	*P*-value	*r*	Change at 24 weeks median (IQR)	*P*-value	
Q1 Current treatment	0.00 (0.00 to 1.00)	.22	0.37	0.00 (0.00 to 1.00)	.40	0.26
Q2 Frequency of hyperglycemia	0.00 (0.00 to 1.00)	.46	0.22	0.00 (−1.00 to 1.00)	.89	−0.04
Q3 Frequency of hypoglycemia	0.00 (0.00 to 2.00)	.17	0.42	0.00 (−1.00 to 1.00)	.72	−0.11
Q4 Convenience	1.00 (0.00 to 2.00)	**.02**	0.72	1.00 (0.00 to 3.00)	.06	0.57
Q5 Flexibility	1.00 (0.00 to 2.00)	**.047**	0.60	1.00 (0.00 to 2.00)	.11	0.48
Q6 Understanding	1.00 (0.00 to 1.00)	.14	0.45	0.00 (0.00 to 1.00)	.13	0.46
Q7 Recommend	0.00 (0.00 to 0.00)	.60	−0.16	0.00 (0.00 to 0.00)	.83	−0.06
Q8 Continue	0.00 (0.00 to 0.00)	.92	0.03	0.00 (0.00 to 1.00)	.69	0.12

CI, confidence interval; SD, standard deviation; MAGE, mean amplitude of glycemic excursions; LBGI, low blood glucose index; SMBG, self-monitoring of blood glucose; DTSQ, Diabetes Treatment Satisfaction Questionnaire. *P*-values <.05 are shown in bold.

[a] n = 10

Supplementary Table S7. Effect sizes of glycemic outcomes and questionnaire scores in patients whose diabetes treatment was reduced at 24 weeks (n = 15).

	Change at 12 weeks (95% CI)	*P*-value	Cohen's d (95% CI)	Change at 24 weeks (95% CI)	*P*-value	Cohen's d (95% CI)
HbA1c (%)	−0.00 (−0.42 to 0.42)	1.00	0.00 (−0.72 to 0.72)	−0.16 (−0.54 to 0.22)	.38	−0.19 (−0.91 to 0.53)
Insulin dose (Units per day)	−2.00 (−3.81 to −0.19)	**.03**	−0.11 (−0.83 to 0.61)	−3.60 (−5.90 to −1.30)	**.01**	−0.20 (−0.91 to 0.52)
**Glycemic outcome**						
SD of glucose (mg/dL)	4.60 (−6.53 to 15.73)	.39	0.15 (−0.56 to 0.87)	−4.88 (−16.35 to 6.59)	.38	−0.21 (−0.92 to 0.51)
MAGE (mg/dL)	−3.71 (−31.40 to 23.99)	.78	−0.06 (−0.78 to 0.66)	−28.04 (−53.21 to −2.87)	**.03**	−0.56 (−1.28 to 0.18)
LBGI	1.39 (−2.00 to 4.78)	.39	0.27 (−0.45 to 0.99)	−0.08 (−1.74 to 1.58)	.92	−0.02 (−0.74 to 0.69)
Frequency of daily SMBG	0.79 (−0.15 to 1.72)	.09	0.49 (−0.24 to 1.21)	0.49 (−0.09 to 1.07)	.09	0.42 (−0.31 to 1.14)
DTSQ Total score	−0.40 (−2.15 to 1.35)	.63	−0.06 (−0.78 to 0.65)	0.20 (−2.22 to 2.62)	.86	0.03 (−0.68 to 0.75)
**DTSQ score**	Change at 12 weeks median (IQR)	*P*-value	*r*	Change at 24 weeks median (IQR)	*P*-value	*r*
Q1 Current treatment	0.00 (0.00 to 0.00)	.08	0.45	0.00 (−1.00 to 0.00)	.16	−0.37
Q2 Frequency of hyperglycemia	−1.00 (−3.00 to 0.00)	.09	−0.44	−1.00 (−2.00 to 0.00)	**.047**	−0.51
Q3 Frequency of hypoglycemia	0.00 (−1.00 to 0.00)	.31	−0.26	0.00 (−2.00 to 2.00)	.93	−0.02
Q4 Convenience	0.00 (−1.00 to 0.00)	.76	−0.08	0.00 (−1.00 to 2.00)	.54	0.16
Q5 Flexibility	0.00 (0.00 to 1.00)	.25	0.30	0.00 (0.00 to 2.00)	.18	0.34
Q6 Understanding	0.00 (0.00 to 0.00)	.56	0.15	0.00 (0.00 to 1.00)	.38	0.23
Q7 Recommend	0.00 (−1.00 to 0.00)	.05	−0.50	0.00 (−1.00 to 0.00)	.23	−0.31
Q8 Continue	0.00 (0.00 to 0.00)	.32	−0.26	0.00 (−1.00 to 0.00)	.43	−0.21

CI, confidence interval; SD, standard deviation; MAGE, mean amplitude of glycemic excursions; LBGI, low blood glucose index; SMBG, self-monitoring of blood glucose; MDI, multiple daily injections; DTSQ, Diabetes Treatment Satisfaction Questionnaire. *P*-values <.05 are shown in bold.
